# Dr. Harry C. Jones

**Published:** 1916-05

**Authors:** 


**Dr. Harry C. Jones,** Prof. of Physical Chemistry at Johns Hopkins, committed suicide by cyanid poisoning, April 9. He was in a bad state of health.

